# Supplementary material for: MAEL Augments Cancer Stemness Properties and Resistance to Sorafenib in Hepatocellular Carcinoma through the PTGS2/AKT/STAT3 Axis
Source: Cancers (Basel). 2022 Jun 10;14(12):2880. doi: 10.3390/cancers14122880 (PMC9221398; doi:10.3390/cancers14122880)
Supplement: Supplementary file 1 [file cancers-14-02880-s001.zip › cancers-1699648-supplementary.pdf]

cancers

Supplementary Material: MAEL Augments Cancer Stemness Properties and Resistance to Sorafenib in Hepatocellular Carcinoma through the PTGS2/AKT/STAT3 Axis

Chaoran Shi, Dora Lai-Wan Kwong, Xue Li, Xia Wang, Xiaona Fang, Liangzhan Sun, Ying Tang, Xin-Yuan Guan and Shan-Shan Li

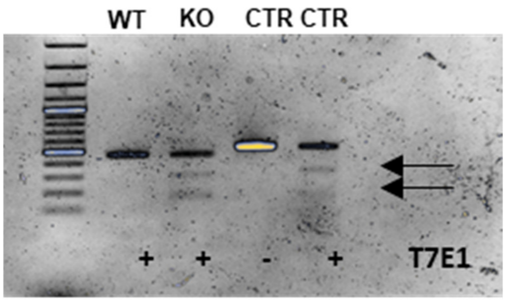

Figure S1. Representative image of T7E1 assay of DNA mismatch detection in MAEL knockout cells. DNA mismatch in MAEL knockout was confirmed as two cut bands were observed. Lanes from left to right: DNA ladder, WT: MAEL wild type amplicon, KO: MAEL knockout amplicon, CTR: Control mismatch DNA product with (CTR) without or with T7E1 digestion.

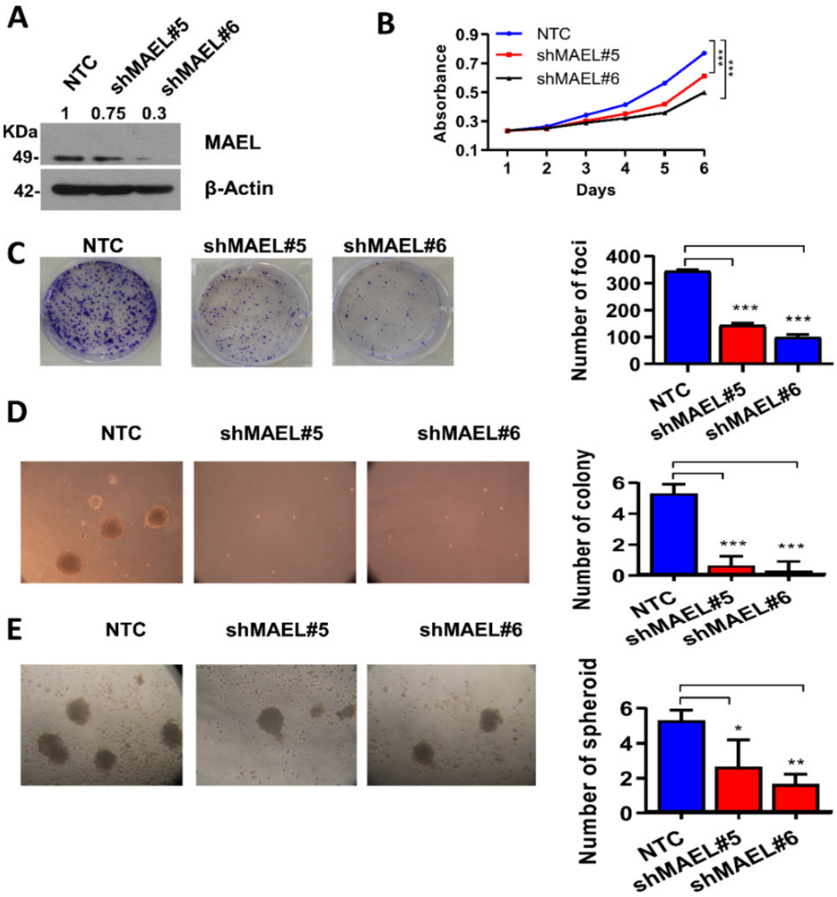

Figure S2. MAEL silence suppressed oncogenic features in PLC8024 cells. (**A**) Western Blots indicates MAEL expression in 8024 cells transfected with shRNA scramble (NTC), #5 and #6 MAEL

shRNA. β-Actin served as internal control. Full Western Blot can be found in Figure S11. (**B**) Growth curve of PLC8024 cells with or without MAEL silence. Representative images and quantitation of (**C**) foci formation, (**D**) colony formation and (**E**) spheroid formation in PLC8024 cells transfected with shRNA scramble or MAEL shRNA. The values indicate the mean ± SD of three independent experiments (* $p < 0.05$; ** $p < 0.01$; *** $p < 0.001$).

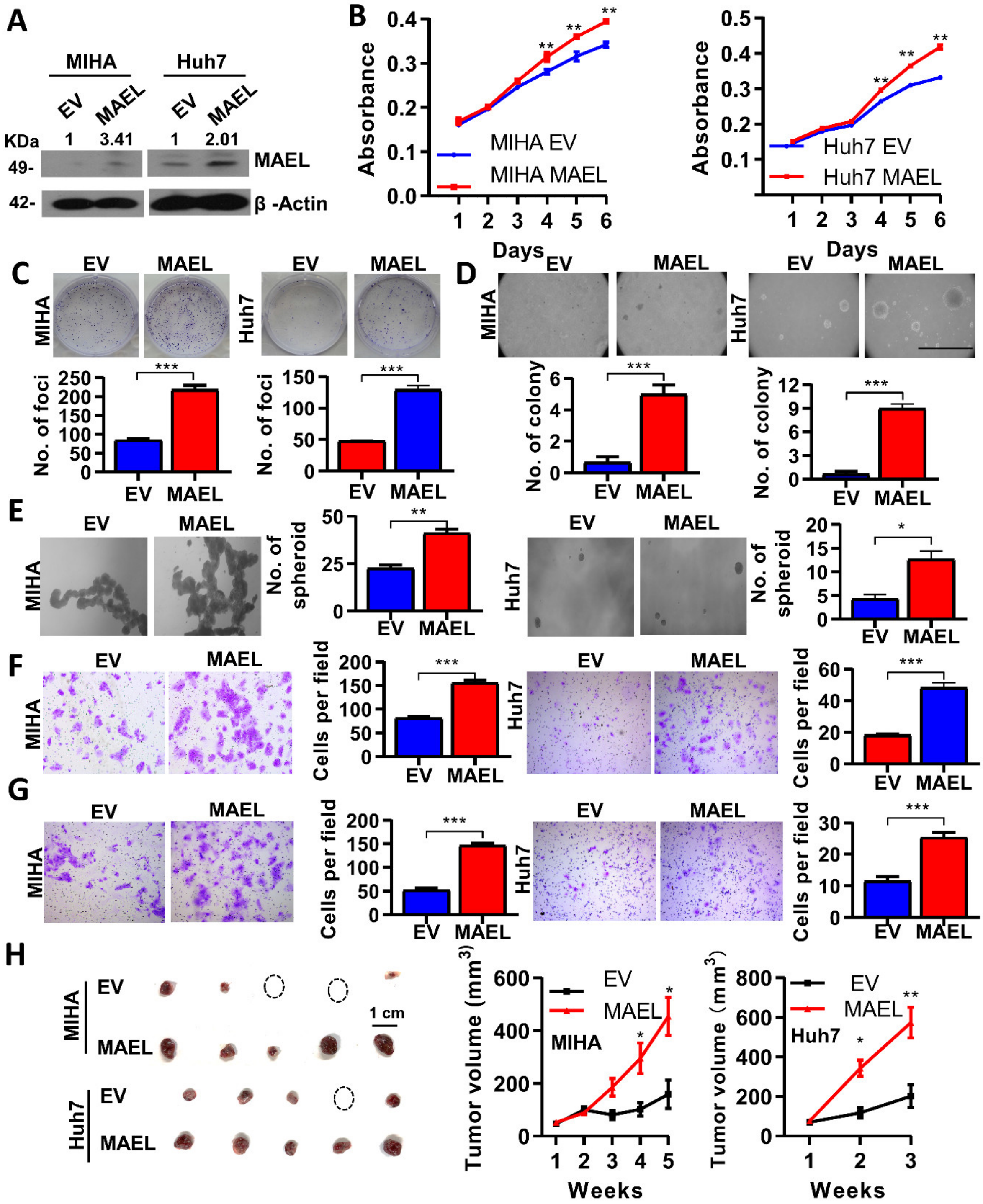

Figure S3. MAEL positively regulates aggressive properties in HCC. (**A**) Western Blots showed ectopic expression of MAEL in MIHA and Huh7 cells. β-Actin served as internal control. Full Western Blot can be found in Figure S12. (**B**) Growth curve of MAEL stable transfected cells determined by XTT assay. Representative images and quantitation of (**C**) foci formation, colony formation in soft

agar (**D**) and (**E**) spheroids formation in MIHA and Huh7 cells with or without MAEL modulated. Representative images and quantitation of (**F**) migrated and (**G**) invaded cells in MIHA and Huh7 cells with or without MAEL modulated. (**H**) Representative images and tumor volumes of xenograft tumors of MIHA and Huh7 cells with or without MAEL overexpression ($n = 5$ per group). EV, empty vector control. The circles stand for no tumor formatted. The values indicate the mean ± SD of three independent experiments. * $p < 0.05$; ** $p < 0.01$; *** $p < 0.001$.

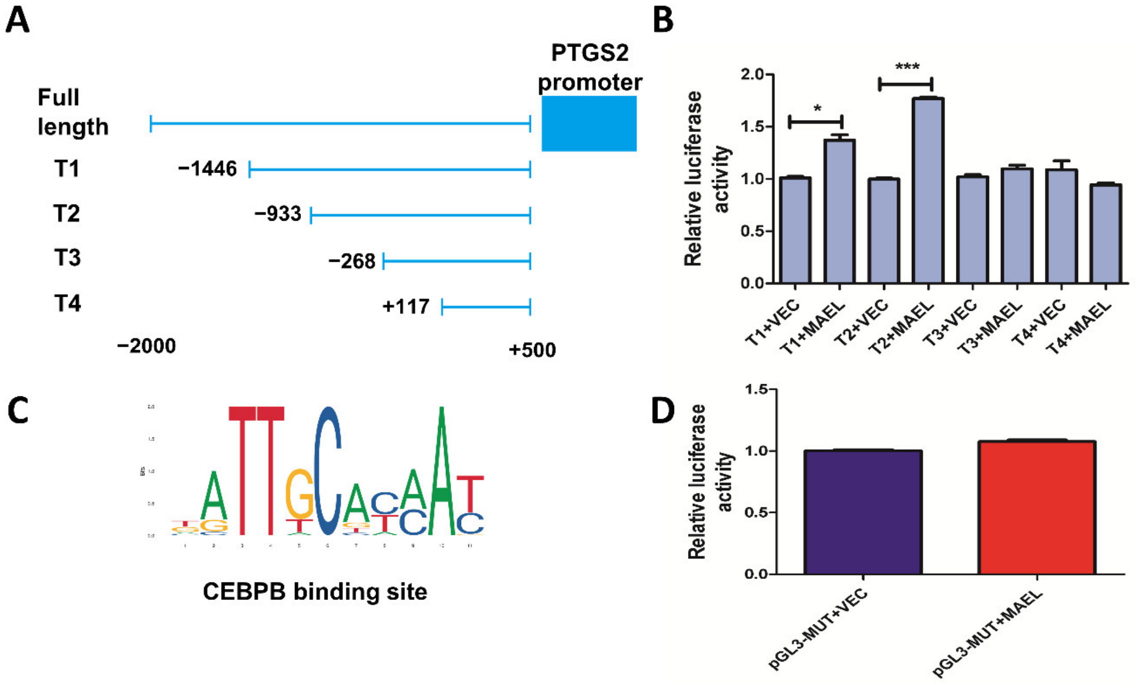

Figure S4. MAEL induces PTGS2 expression via CEBPB. (**A**) Schematic illustrator of the different lengths of truncated mutants of PTGS2 promoter. (**B**) Relative luciferase activity of the truncated mutants T1~T4 within PTGS2 promoter region. (**C**) The candidate binding sites CEBPB sequence predicted by JASPER. (**D**) Relative luciferase activity after CEBPB binding site mutation. The values indicate the mean ± SD of three independent experiments. * $p < 0.05$; *** $p < 0.001$.

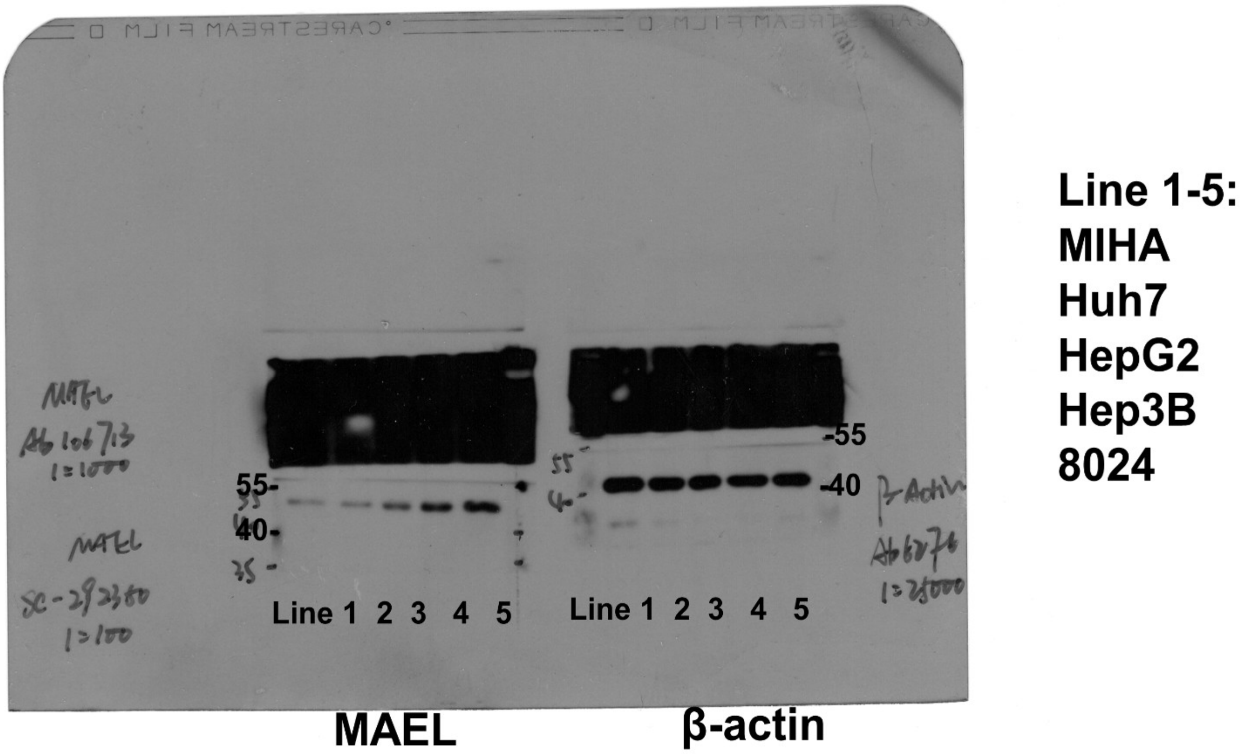

Figure S5. Uncropped Western Blot from Figure 1D.

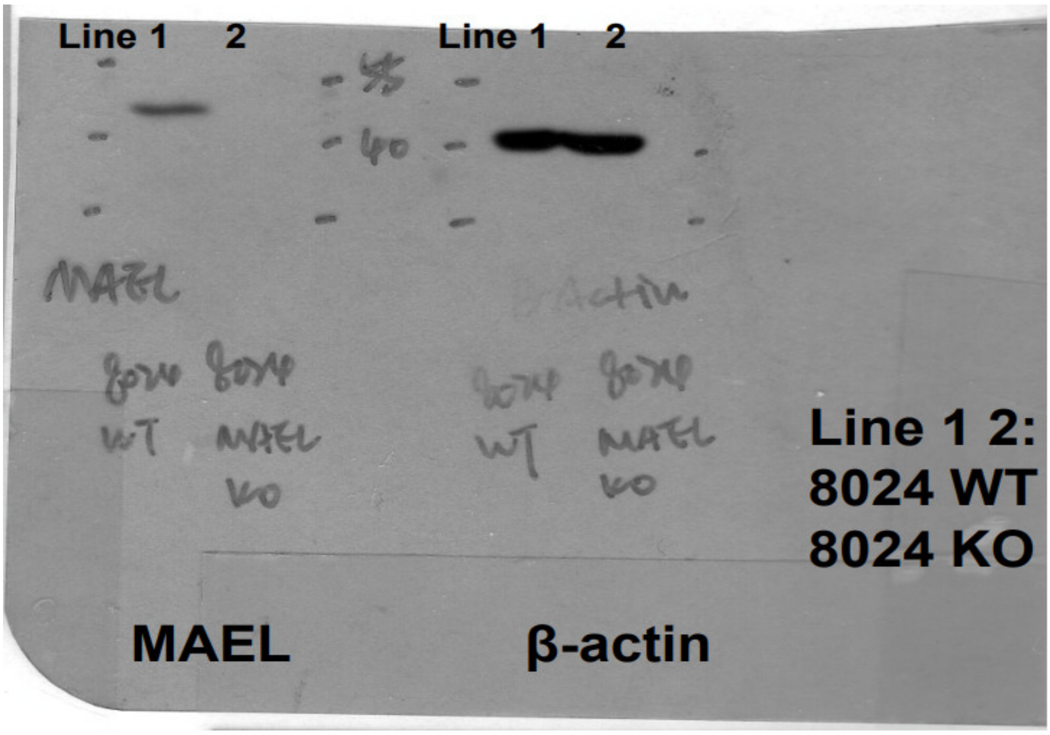

Figure S6. Uncropped Western Blot from Figure 2A.

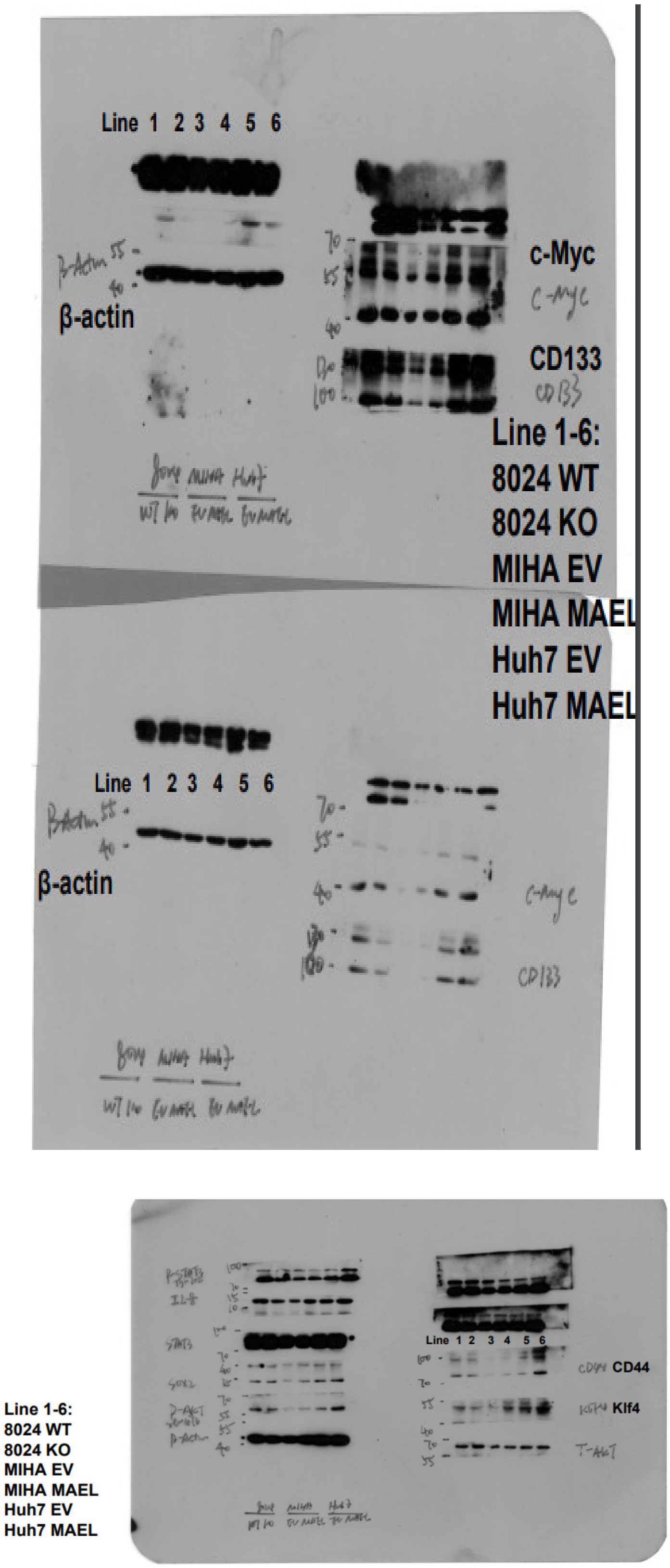

Figure S7. Uncropped Western Blot from Figure 3B.

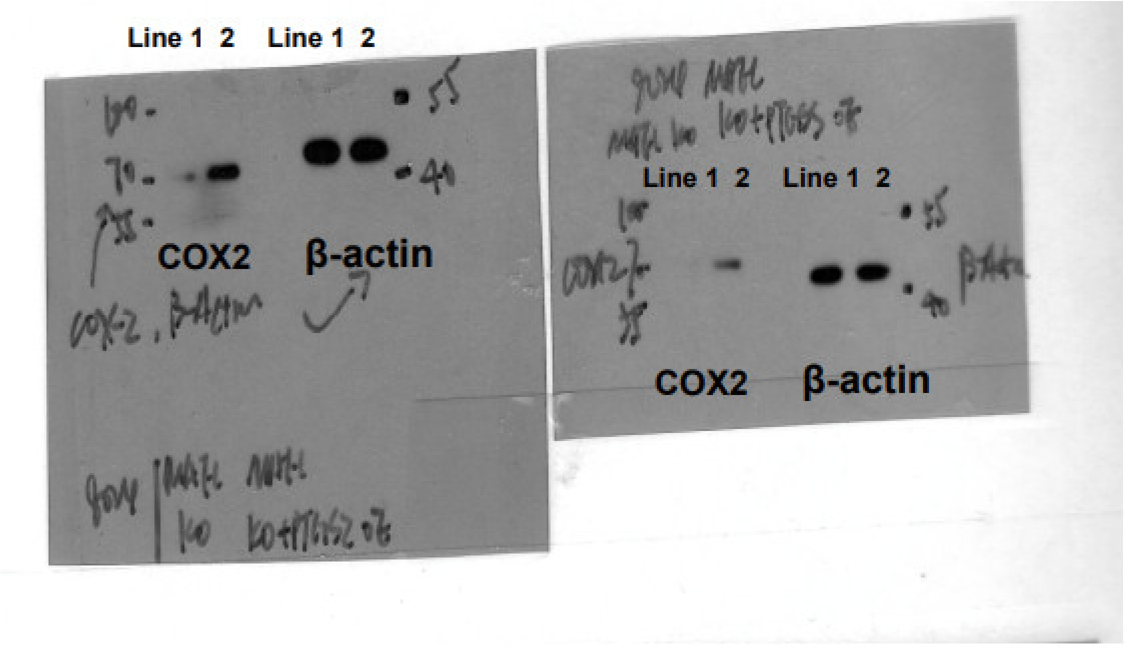

Line 1-2:
8024 KO+EV
8024 KO+PTGS2

Figure S8. Uncropped Western Blot from Figure 6A.

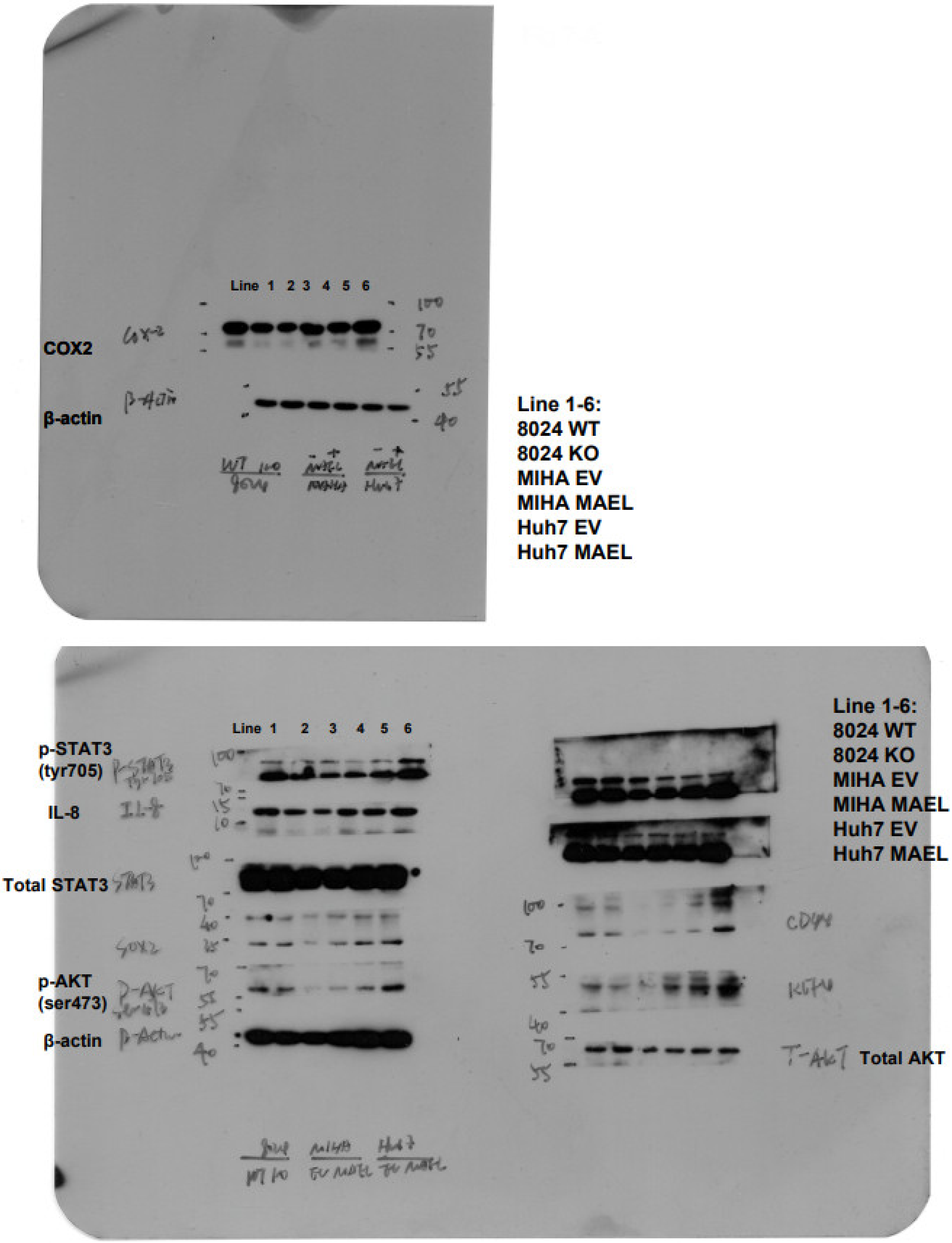

Figure S9. Uncropped Western Blot from Figure 7A.

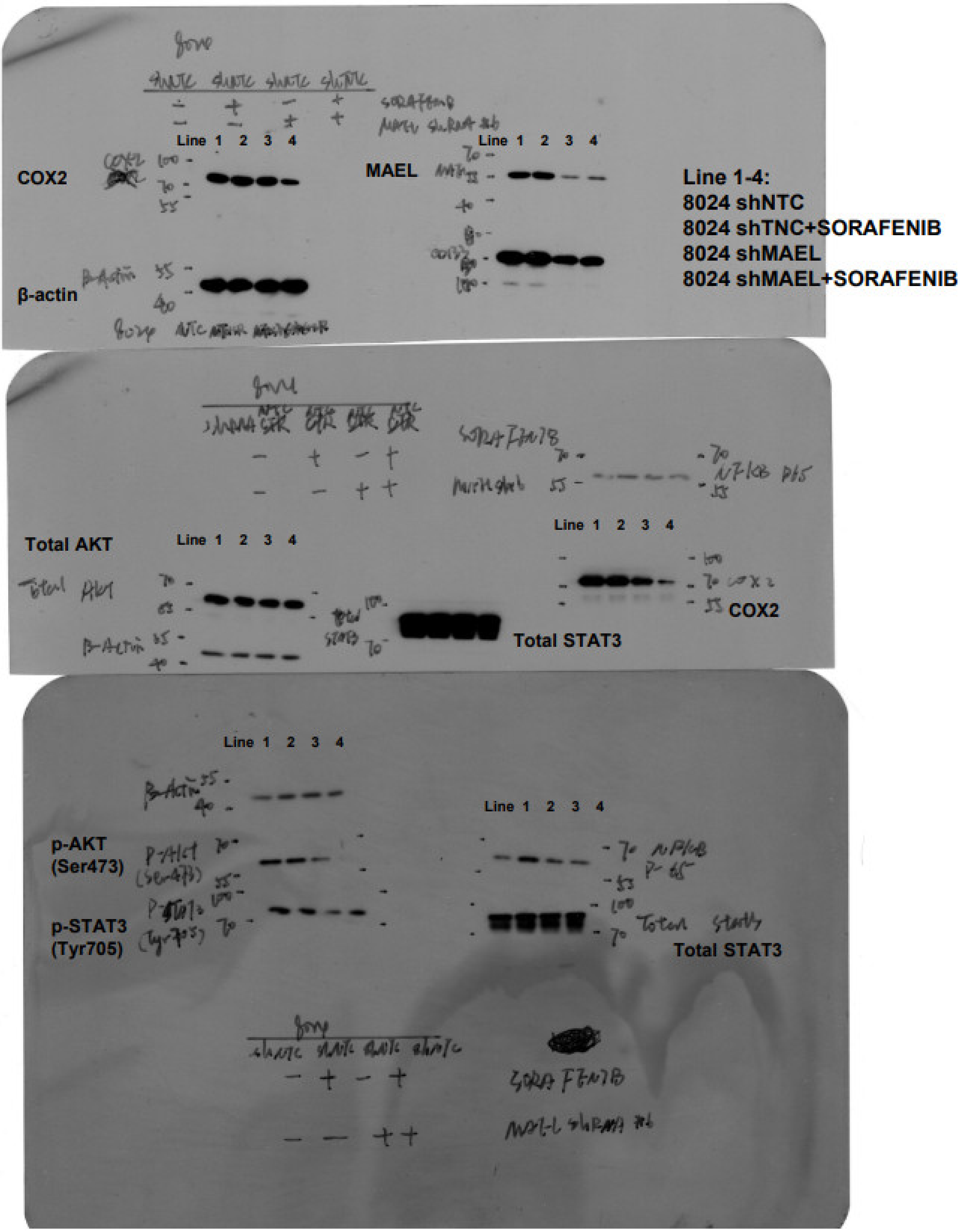

Figure S10. Uncropped Western Blot from Figure 7D.

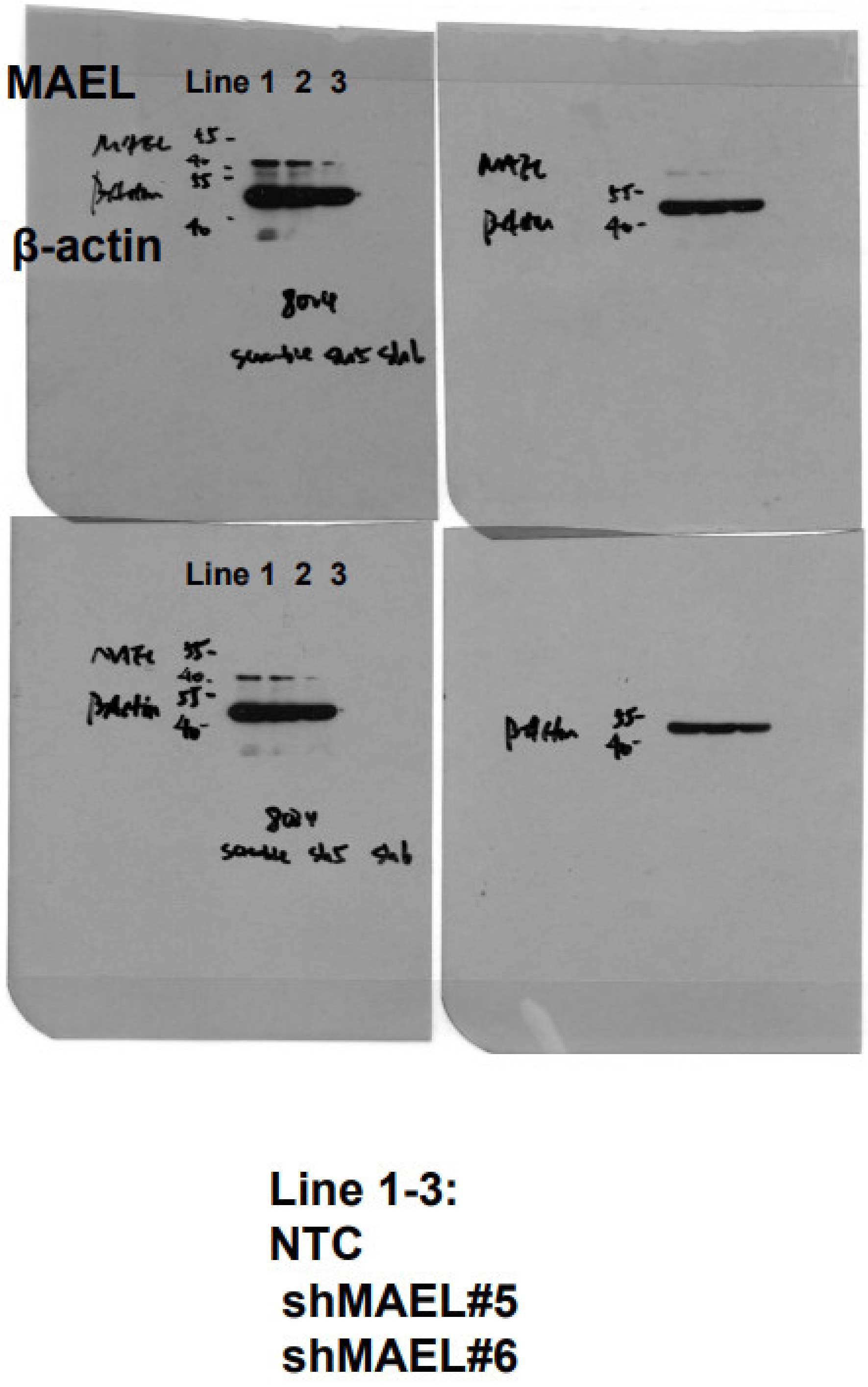

Figure S11. Uncropped Western Blot from Figure S2A.

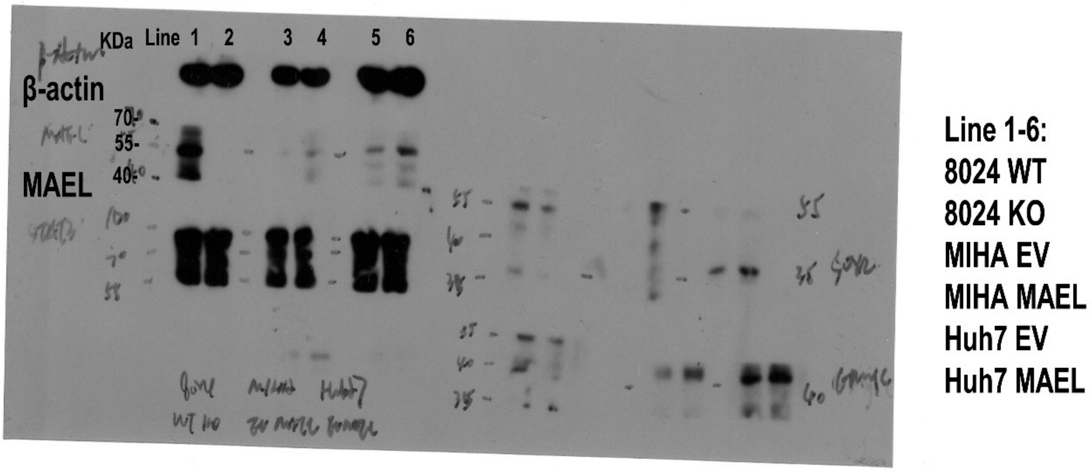

Figure S12. Uncropped Western Blot from Figure S3A.

Table S1. Primer list.

Gene	Primers Forward Primer (5′ to 3′)	Reverse Primer (5′ to 3′)
CD133	TGGATGCAGAACTTGACAACGT	ATACCTGCTACGACAGTCGTGGT
CD44	TTGCAGTCAACAGTCGAAGAAG	CCTTGTTCACCAAATGCACCA
Oct4	CTTGCTGCAGAAGTGGGTGGAGGAA	CTGCAGTGTGGGTTTCGGGCA
MAEL	CCACGAGGATTTCGATTTCATT	ACAGTTGCTTGGTTATGCCCAC
Myc	GGCTCCTGGCAAAAGGTCA	CTGCGTAGTTGTGCTGATGT
SOX2	GCCGAGTGGAAACTTTTGTCG	GGCAGCGTGTACTTATCCTTCT
Nanog	AATACCTCAGCCTCCAGCAGATG	TGCGTCACACCATTGCTATTCTTC
Epcam	AATCGTCAATGCCAGTGTACTT	TCTCATCGCAGTCAGGATCATAA
18s	TCGGTCTTATTTTGCTGGTTT	ATCGCTAGTTGGCATCGTTT
MAEL Clone	CACCAATCTTCCAGTCTCAGGCTGTTTGT	GGAAGCAGAACAATCCCTCAAGAAA
PTGS2 Clone	CACCGCCGTGGCCGCCGCCGCGATG	AGACTTCTACAGTTCAGTCGAACG
PTGS2 promoter	GCTAGCTCTTCATCGCCTTCACAG	CTCGAGTCCACAGATCCCTCAAAA
PTGS2 T1	CAGTTCTTTCCTGCTCCC	CTCGAGTCCACAGATCCCTCAAAA
PTGS2 T2	AAATCCTTGCTGTTCCCACC	CTCGAGTCCACAGATCCCTCAAAA
PTGS2 T3	GGTGCCATACTCAGCCATTC	CTCGAGTCCACAGATCCCTCAAAA
PTGS2 T4	CCTTCCTCCTGTGCCTGAT	CTCGAGTCCACAGATCCCTCAAAA
PTGS2 Mut	TTGGACCGCTAGAGTTCG	AGACGAAGAAAGGGAGGG

Table S2. Antibody list.

Antibody	Host	Dilution	Source/Vendor	Catalog
β-Actin	Mouse	1:20000	Abcam	ab6276
MAEL	Mouse	1:100	Santa Cruz	sc-398925
SOX2	Rabbit	1:1000	Abcam	ab97959
c-Myc	Mouse	1:1000	Invitrogen	13-2500
Klf4	Rabbit	1:1000	Abcam	ab215036
IL8	Rabbit	1:1000	Abcam	ab110727
CD133	Rabbit	1:1000	Abcam	ab216323
CD44	Mouse	1:1000	Cell signaling technology	CST 3570
NF-κB p65 (L8F6)	Mouse	1:1000	Cell signaling technology	CST 6956
Akt	Rabbit	1:1000	Cell signaling technology	CST 4691
p-Akt (Ser473)	Rabbit	1:1000	Cell signaling technology	CST 9271
STAT3	Mouse	1:1000	Cell signaling technology	CST 9139
p-STAT3 (Tyr 705)	Rabbit	1:1000	Cell signaling technology	CST 9145
COX-2	Rabbit	1:200	Santa Cruz	sc-376861
HRP-linked anti-Mouse IgG	Horse	1:5000	Cell signaling technology	CST 7076
HRP-linked anti-Rabbit IgG	Goat	1:2000	Cell signaling technology	CST 7074

PE-CD133	Mouse	1:100	Miltenyi Biotec	130-120-145
PE-IgG1 kappa Isotype Control	Mouse	1:100	Invitrogen	12-4714-82
Goat anti-Mouse IgG (H+L)-Alexa Fluor® 555	Rabbit	1:100	Invitrogen	21424

Table S3. Correlation between MAEL expression and clinicopathological in TCGA-LIHC cohort.

Variables	Sum	MEAL High Expression (185)	MAEL Low Expression (186)	*P* Value
Age (years)				
AGE ≤60	149	74	75	
AGE >60	201	111	90	0.302908
AFP (ng/mL)				
≤20	240	117	123	
>20	131	68	63	0.56099
Ishak fibrosis score				
≤0	74	35	39	
1–6	138	63	75	0.81886
Gender				
Male	184	124	60	
Female	187	61	126	<0.00001
Tumor staging (AJCC)				
Stage I–II	257	131	126	
Stage III–IV	90	39	51	0.212149
Hepatitis B virus surface Ag				
Positive	139	74	65	
Negative	234	111	123	0.278567
Tumor grade				
Grade I-II	231	116	115	
Grade III-IV	134	67	67	0.9682

Statistical analysis was based on available clinicopathological data.
